# 2,3,4-Tribromo­thio­phene

**DOI:** 10.1107/S1600536808006600

**Published:** 2008-03-14

**Authors:** Tony M. Kuriger, Stephen C. Moratti, Jim Simpson

**Affiliations:** a Department of Chemistry, University of Otago, PO Box 56, Dunedin, New Zealand

## Abstract

In the title compound, C_4_HBr_3_S, there are two essentially planar mol­ecules in the asymmetric unit. In the crystal structure, bifurcated C—H⋯Br hydrogen bonds link the mol­ecules into chains. Weak Br⋯Br inter­actions [Br⋯Br = 3.634 (4)–3.691 (4) Å] then lead to undulating sheets in the *bc* plane.

## Related literature

For related polybromo­thio­phene structures, see: Helmholdt *et al.* (2007 ▶); Murakami *et al.* (2002 ▶); Xie *et al.* (1997 ▶, 1998 ▶). For information on halogen⋯halogen contacts, see: Pedireddi *et al.* (1994 ▶). For details of the Cambridge Structural Database, see: Allen (2002 ▶).
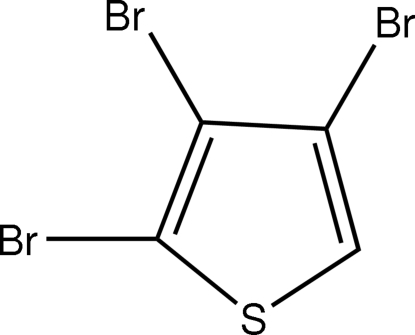


## Experimental

### 

#### Crystal data

C_4_HBr_3_S
*M* *_r_* = 320.84
Orthorhombic, 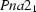

*a* = 12.4529 (11) Å
*b* = 3.9724 (4) Å
*c* = 28.846 (3) Å
*V* = 1426.9 (2) Å^3^
*Z* = 8
Mo *K*α radiation
μ = 17.14 mm^−1^
*T* = 91 (2) K
0.17 × 0.06 × 0.02 mm

#### Data collection

Bruker APEXII CCD area-detector diffractometer
Absorption correction: multi-scan (*SADABS*; Bruker, 2006 ▶) *T*_min_ = 0.434, *T*_max_ = 0.710
12082 measured reflections
2163 independent reflections
1852 reflections with *I* > 2σ(*I*)
*R*_int_ = 0.092
θ_max_ = 23.7°

#### Refinement

*R*[*F* ^2^ > 2σ(*F* ^2^)] = 0.061
*wR*(*F* ^2^) = 0.172
*S* = 0.86
2163 reflections
109 parameters
1 restraint
H-atom parameters constrained
Δρ_max_ = 3.39 e Å^−3^
Δρ_min_ = −1.30 e Å^−3^
Absolute structure: Flack (1983 ▶), 1050 Friedel pairs
Flack parameter: 0.11 (6)

### 

Data collection: *APEX2* (Bruker 2006 ▶); cell refinement: *APEX2* and *SAINT* (Bruker 2006 ▶); data reduction: *SAINT*; program(s) used to solve structure: *SHELXS97* (Sheldrick, 2008 ▶) and *TITAN* (Hunter & Simpson, 1999 ▶); program(s) used to refine structure: *SHELXL97* (Sheldrick, 2008 ▶) and *TITAN*; molecular graphics: *ORTEP-3* (Farrugia, 1997 ▶) and *Mercury* (Macrae *et al.*, 2006 ▶); software used to prepare material for publication: *SHELXL97*, *enCIFer* (Allen *et al.*, 2004 ▶) and *PLATON* (Spek, 2003 ▶).

## Supplementary Material

Crystal structure: contains datablocks I, global. DOI: 10.1107/S1600536808006600/hb2706sup1.cif

Structure factors: contains datablocks I. DOI: 10.1107/S1600536808006600/hb2706Isup2.hkl

Additional supplementary materials:  crystallographic information; 3D view; checkCIF report

## Figures and Tables

**Table 1:** Hydrogen-bond geometry (Å, °)

*D*—H⋯*A*	*D*—H	H⋯*A*	*D*⋯*A*	*D*—H⋯*A*
C1*A*—H1*A*⋯Br3*A*^i^	0.95	3.04	3.89 (3)	149
C1*A*—H1*A*⋯Br4*A*^i^	0.95	2.96	3.68 (3)	134
C1*B*—H1*B*⋯Br3*B*^ii^	0.95	2.93	3.79 (3)	151
C1*B*—H1*B*⋯Br4*B*^ii^	0.95	2.97	3.66 (2)	131

Symmetry codes: (i) 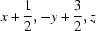; (ii) 
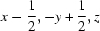
.

**Fig. 1.**
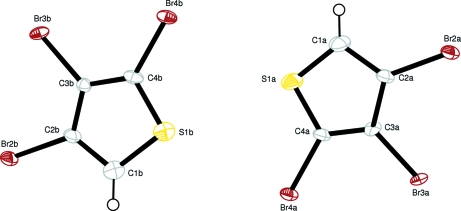
The asymmetric unit of (I), with 50% displacement ellipsoids for the non-hydrogen atoms.

**Fig. 2.**
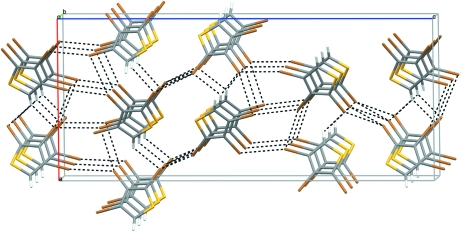
Crystal packing of (I) with C—H···Br hydrogen bonds and Br···Br interactions drawn as dashed lines.

## supplementary crystallographic information

### Comment

Brominated thiophenes are very important intermediates in the construction of thiophene oligomers and polymers for use in optoelectronics. In some cases, it is important to have one or two α-positions free for further oxidative coupling. The 2,3,4-tribromo derivative is not easy to access, as the 2- and 5-positions are normally substituted first, and so it is normally synthesized *via* debromination from tetrabromothiophene (Xie *et al.*, 1998).

The asymmetric unit of the title compound, (I), C_8_H_2_Br_6_S_2_, consists of two discrete tribromothiophene molecules A & B (Fig. 1). Each molecule is essentially planar with r.m.s. deviations from the mean planes through all non-hydrogen atoms of 0.0194 and 0.0286 Å for A and B respectively. The dihedral angle between the A and B ring planes is 0.9 (4)° but they are well separated with a centroid to centroid distance of 6.3 Å.

In the crystal of (I) bifurcated C—H···Br hydrogen bonds (Table 1) form chains of like molecules that pack in an obverse fashion along a. The structure is further stabilized by an extensive network of weak Br···Br interactions with Br···Br distances in the range 3.634 (4)Å (Br3A···Br2B^i^, i = 1 - *x*, 1 - *y*, -1/2 + *z*; θ_1_ = 156.7° and θ_2_ = 117.5°) (Pedireddi *et al.*, 1994) to 3.691 (4)Å (Br3A···Br2A^ii^ ii = -1/2 + *x*, 1/2 - *y*, *z*; θ_1_ = 161.8° and θ_2_ = 84.7°). These contacts link the chains of molecules into undulating sheets in the *bc* plane (Fig. 2).

### Experimental

2,3,4-Tribromothiophene, prepared by the method of Xie *et al.* (1998), was dissolved in methanol. Colourless plates of (I) were grown by slow diffusion of water into the solution.

### Refinement

The crystals were small and very weakly diffracting and little data were obtainable beyond θ = 23°. This clearly contributes to the relatively high *R* factor and poor precision of the data in this determination. The C-bound H atoms were placed geometrically (C—H = 0.95 Å) and refined as riding with *U*_iso_(H) = 1.2*U*_eq_(C). A number of high peaks were found in the final difference map in the vicinity of the Br atoms in both molecules. The deepest hole is 0.98Å from Br3B.

### Crystal data

**Table:**

C_4_HBr_3_S	*F*_000_ = 1168
*M**_r_* = 320.84	*D*_x_ = 2.987 Mg m^−^^3^
Orthorhombic, *P**n**a*2_1_	Mo *K*α radiation λ = 0.71073 Å
Hall symbol: P 2c -2n	Cell parameters from 1448 reflections
*a* = 12.4529 (11) Å	θ = 3.4–21.9º
*b* = 3.9724 (4) Å	µ = 17.14 mm^−^^1^
*c* = 28.846 (3) Å	*T* = 91 (2) K
*V* = 1426.9 (2) Å^3^	Plate, colourless
*Z* = 8	0.17 × 0.06 × 0.02 mm

### Data collection

**Table:**

Bruker APEXII CCD area-detector diffractometer	2163 independent reflections
Radiation source: fine-focus sealed tube	1852 reflections with *I* > 2σ(*I*)
Monochromator: graphite	*R*_int_ = 0.092
*T* = 91(2) K	θ_max_ = 23.7º
ω scans	θ_min_ = 1.4º
Absorption correction: multi-scan(SADABS; Bruker, 2006)	*h* = −14→14
*T*_min_ = 0.434, *T*_max_ = 0.710	*k* = −4→4
12082 measured reflections	*l* = −32→32

### Refinement

**Table:**

Refinement on *F*^2^	Hydrogen site location: inferred from neighbouring sites
Least-squares matrix: full	H-atom parameters constrained
*R*[*F*^2^ > 2σ(*F*^2^)] = 0.061	*w* = 1/[σ^2^(*F*_o_^2^) + (0.1079*P*)^2^ + 95.665*P*] where *P* = (*F*_o_^2^ + 2*F*_c_^2^)/3
*wR*(*F*^2^) = 0.172	(Δ/σ)_max_ = 0.001
*S* = 0.86	Δρ_max_ = 3.39 e Å^−^^3^
2163 reflections	Δρ_min_ = −1.30 e Å^−^^3^
109 parameters	Extinction correction: none
1 restraint	Absolute structure: Flack (1983), 1050 Friedel pairs
Primary atom site location: structure-invariant direct methods	Flack parameter: 0.11 (6)
Secondary atom site location: difference Fourier map	

### Special details

**Table:**

Experimental. As the crystals were small and very weakly diffracting, data were collected using 55 sec exposures per frame.
Geometry. All e.s.d.'s (except the e.s.d. in the dihedral angle between two l.s. planes) are estimated using the full covariance matrix. The cell e.s.d.'s are taken into account individually in the estimation of e.s.d.'s in distances, angles and torsion angles; correlations between e.s.d.'s in cell parameters are only used when they are defined by crystal symmetry. An approximate (isotropic) treatment of cell e.s.d.'s is used for estimating e.s.d.'s involving l.s. planes.
Refinement. Refinement of *F*^2^ against ALL reflections. The weighted *R*-factor *wR* and goodness of fit *S* are based on *F*^2^, conventional *R*-factors *R* are based on *F*, with *F* set to zero for negative *F*^2^. The threshold expression of *F*^2^ > σ(*F*^2^) is used only for calculating *R*-factors(gt) *etc*. and is not relevant to the choice of reflections for refinement. *R*-factors based on *F*^2^ are statistically about twice as large as those based on *F*, and *R*- factors based on ALL data will be even larger.

### Fractional atomic coordinates and isotropic or equivalent isotropic displacement parameters (Å^2^)

**Table:**

	*x*	*y*	*z*	*U*_iso_*/*U*_eq_	
S1A	0.6553 (5)	0.6506 (19)	0.2528 (2)	0.0307 (15)	
C1A	0.723 (2)	0.726 (7)	0.2042 (10)	0.0307 (15)	
H1A	0.7916	0.8311	0.2015	0.037*	
C2A	0.6527 (19)	0.592 (6)	0.1649 (8)	0.0229 (6)	
Br2A	0.69172 (17)	0.6009 (6)	0.10260 (10)	0.0229 (6)	
C3A	0.5527 (17)	0.441 (7)	0.1819 (9)	0.021 (5)	
Br3A	0.44805 (17)	0.2573 (6)	0.14378 (10)	0.0183 (7)	
C4A	0.5485 (18)	0.455 (6)	0.2298 (8)	0.0197 (6)	
Br4A	0.43447 (16)	0.3131 (7)	0.26658 (9)	0.0197 (6)	
S1B	0.6092 (5)	0.3531 (17)	0.3764 (2)	0.0268 (14)	
C1B	0.542 (2)	0.270 (6)	0.4273 (10)	0.0268 (14)	
H1B	0.4743	0.1617	0.4311	0.032*	
C2B	0.6136 (18)	0.407 (7)	0.4637 (8)	0.0227 (6)	
Br2B	0.57498 (17)	0.4088 (6)	0.52692 (10)	0.0227 (6)	
C3B	0.7118 (17)	0.544 (6)	0.4479 (8)	0.016 (5)	
Br3B	0.82097 (17)	0.7329 (6)	0.48587 (10)	0.0193 (7)	
C4B	0.7212 (17)	0.523 (6)	0.4001 (8)	0.0206 (6)	
Br4B	0.83237 (18)	0.6820 (7)	0.36312 (9)	0.0206 (6)	

### Atomic displacement parameters (Å^2^)

**Table:**

	*U*^11^	*U*^22^	*U*^33^	*U*^12^	*U*^13^	*U*^23^
S1A	0.017 (3)	0.039 (4)	0.037 (4)	0.000 (3)	−0.004 (3)	0.002 (3)
C1A	0.017 (3)	0.039 (4)	0.037 (4)	0.000 (3)	−0.004 (3)	0.002 (3)
C2A	0.0147 (12)	0.0294 (16)	0.0246 (13)	−0.0022 (10)	0.0041 (10)	0.0019 (12)
Br2A	0.0147 (12)	0.0294 (16)	0.0246 (13)	−0.0022 (10)	0.0041 (10)	0.0019 (12)
C3A	0.005 (10)	0.035 (14)	0.024 (13)	0.008 (10)	−0.002 (9)	−0.006 (12)
Br3A	0.0104 (11)	0.0209 (16)	0.0237 (16)	−0.0040 (9)	−0.0040 (10)	−0.0015 (9)
C4A	0.0138 (12)	0.0212 (11)	0.0243 (14)	−0.0028 (9)	0.0055 (9)	−0.0012 (14)
Br4A	0.0138 (12)	0.0212 (11)	0.0243 (14)	−0.0028 (9)	0.0055 (9)	−0.0012 (14)
S1B	0.023 (3)	0.023 (3)	0.034 (4)	0.004 (3)	0.001 (3)	−0.005 (3)
C1B	0.023 (3)	0.023 (3)	0.034 (4)	0.004 (3)	0.001 (3)	−0.005 (3)
C2B	0.0142 (11)	0.0305 (15)	0.0235 (13)	−0.0007 (10)	0.0037 (10)	0.0040 (12)
Br2B	0.0142 (11)	0.0305 (15)	0.0235 (13)	−0.0007 (10)	0.0037 (10)	0.0040 (12)
C3B	0.016 (11)	0.015 (11)	0.017 (12)	0.000 (9)	0.000 (9)	0.002 (10)
Br3B	0.0090 (11)	0.0200 (16)	0.0290 (17)	0.0022 (10)	−0.0020 (10)	−0.0028 (10)
C4B	0.0125 (11)	0.0198 (10)	0.0295 (15)	0.0030 (10)	0.0056 (10)	0.0023 (14)
Br4B	0.0125 (11)	0.0198 (10)	0.0295 (15)	0.0030 (10)	0.0056 (10)	0.0023 (14)

### Geometric parameters (Å, °)

**Table:**

S1A—C4A	1.68 (2)	S1B—C4B	1.69 (2)
S1A—C1A	1.66 (3)	S1B—C1B	1.72 (3)
C1A—C2A	1.53 (4)	C1B—C2B	1.48 (4)
C1A—H1A	0.9500	C1B—H1B	0.9500
C2A—C3A	1.47 (3)	C2B—C3B	1.41 (3)
C2A—Br2A	1.86 (2)	C2B—Br2B	1.89 (2)
C3A—C4A	1.38 (3)	C3B—C4B	1.39 (3)
C3A—Br3A	1.86 (2)	C3B—Br3B	1.90 (2)
C4A—Br4A	1.86 (2)	C4B—Br4B	1.86 (2)
			
C4A—S1A—C1A	98.9 (13)	C4B—S1B—C1B	97.7 (12)
C2A—C1A—S1A	105.5 (17)	C2B—C1B—S1B	103.8 (17)
C2A—C1A—H1A	127.2	C2B—C1B—H1B	128.1
S1A—C1A—H1A	127.2	S1B—C1B—H1B	128.1
C3A—C2A—C1A	112 (2)	C3B—C2B—C1B	116 (2)
C3A—C2A—Br2A	123.5 (18)	C3B—C2B—Br2B	122.1 (17)
C1A—C2A—Br2A	123.9 (18)	C1B—C2B—Br2B	122.1 (18)
C4A—C3A—C2A	110 (2)	C4B—C3B—C2B	112 (2)
C4A—C3A—Br3A	125.6 (19)	C4B—C3B—Br3B	122.4 (17)
C2A—C3A—Br3A	124.0 (19)	C2B—C3B—Br3B	125.8 (17)
C3A—C4A—S1A	112.5 (18)	C3B—C4B—S1B	110.9 (17)
C3A—C4A—Br4A	125.9 (18)	C3B—C4B—Br4B	127.9 (18)
S1A—C4A—Br4A	121.4 (14)	S1B—C4B—Br4B	121.1 (14)

### Hydrogen-bond geometry (Å, °)

**Table:**

*D*—H···*A*	*D*—H	H···*A*	*D*···*A*	*D*—H···*A*
C1A—H1A···Br3A^i^	0.95	3.04	3.89 (3)	149
C1A—H1A···Br4A^i^	0.95	2.96	3.68 (3)	134
C1B—H1B···Br3B^ii^	0.95	2.93	3.79 (3)	151
C1B—H1B···Br4B^ii^	0.95	2.97	3.66 (2)	131

Symmetry codes: (i) *x*+1/2, −*y*+3/2, *z*; (ii) *x*−1/2, −*y*+1/2, *z*.
